# Flash Joule heating for ductilization of metallic glasses

**DOI:** 10.1038/ncomms8932

**Published:** 2015-07-29

**Authors:** I. V. Okulov, I. V. Soldatov, M. F. Sarmanova, I. Kaban, T. Gemming, K. Edström, J. Eckert

**Affiliations:** 1 IFW Dresden, Helmholtzstrasse 20, 01069 Dresden, Germany; 2 TU Dresden, Institut für Werkstoffwissenschaft, 01062 Dresden, Germany; 3 Institute of Natural Sciences, Ural Federal University, 620000 Ekaterinburg, Russia; 4 Leibniz-Institut für Oberflächenmodifizierung, e. V. Permoserstrasse 15, 04318 Leipzig, Germany; 5 Department of Chemistry-Ångström Laboratory, Uppsala University, Box 538, 75121 Uppsala, Sweden

## Abstract

Metallic glasses (MGs) inherit their amorphous structure from the liquid state, which predetermines their ability to withstand high loads approaching the theoretical limit. However, the absence of slip systems makes them very sensitive to the type of loading and extremely brittle in tension. The latter can be improved by precipitation of ductile crystals, which suppress a catastrophic propagation of shear bands in a glassy matrix. Here we report a novel approach to obtain MG-matrix composites with tensile ductility by flash Joule heating applied to Cu_47.5_Zr_47.5_Al_5_ (at.%) metallic glass. This homogeneous, volumetric and controllable rapid heat treatment allows achieving uniformly distributed metastable B2 CuZr crystals in the glassy matrix. It results in a significant tensile strain of 6.8±0.5%. Moreover, optimized adjustment of the heat-treatment conditions enables tuning of microstructure to achieve desired mechanical properties.

## Abstract (web summary)

Metallic glasses (MG) have higher yield strengths than crystalline alloys but at the same time are very brittle, which has hampered practical applications. Here, the authors use flash Joule heating to design a MG-matrix composite with a uniform distribution of ductile crystals, improving mechanical properties.

## Graphical abstract

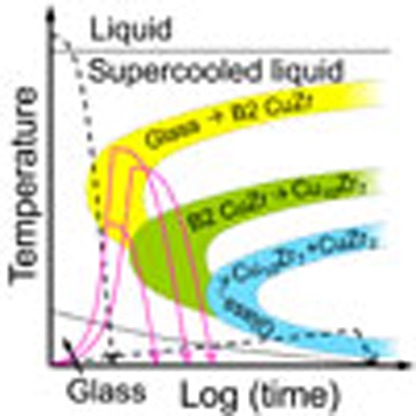

The design of composites aims to achieve a balance of properties, which is superior to either constituent material alone^1^. Among others, metallic glass composites (MGCs) nowadays attract significant technological interest^2^,^3^,^4^,^5^,^6^. The *ex situ*^7^,^8^ and *in situ*^5^,^6^ MGCs consist of crystalline phase(s) embedded in a glassy matrix. Since the glasses inherit their structure directly from liquids up on rapid quenching, they lack the periodic (long range) atomic order and, therefore, exhibit a strength close to the theoretical value^9^. On the other hand, the disordered structure of metallic glasses (MGs) causes their brittle tensile fracture. However, the recent breakthrough discovery of MGs with outstanding fracture toughness^9^,^10^ opens new perspectives for a design of ductile MGCs. This suggests that the size of the plastic zone at a crack tip is the particularly important length scale for an engineering design of fracture-resistant devices from MGs^11^. In other words, to minimize the risk of fast fracture, the dimension of the MG component should not exceed the plastic zone size. This is effectively utilized in micro-electronic devices^9^ and large-scale MGCs^5^,^6^, where the MG phase is sub-divided into fine volumes. Thus, the proper design of MGCs can yield a combination of outstandingly high strength (close to that for MGs) and tensile plasticity (close to that for crystalline materials).

*In situ* MGCs are obtained by casting through an adjustment of composition^12^ and cooling rate^13^. Often, they exhibit a strength close to that of the glassy matrix and acceptable plasticity under uniaxial compression, depending on the volume fraction of crystalline phase(s)^2^,^3^,^6^. However, non-uniform grain size and spatial distribution of as-cast MGCs entails a significant decrease of tensile ductility. Recently, Hofmann *et al*.^5^,^14^ successfully applied semi-solid processing to Vitreloy-type MGs and created MGCs with more uniform microstructure and, therefore, with better tensile plasticity. Unfortunately, that method imposes limitations for the composition and geometry of samples. Moreover, these MGCs suffer from considerable strain softening and necking under tension^15^. The problem of drastic strain softening can be solved by precipitation of crystals, which tend to undergo a martensitic transformation on deformation, for example, the B2 CuZr phase in the CuZr system^4^.

In this work, we propose an advanced Joule heating technique and apply it to amorphous Cu_47.5_Zr_47.5_Al_5_ ribbons for making high-performance MG-matrix composites. The heating rate and processing time are controlled, respectively, by the current density and *in situ* measurement of the resistance of a sample. Variation of the heat-treatment parameters enables obtaining either fully crystalline composites with different volume fraction of B2 CuZr and Cu_10_Zr_7_ phases or composites with uniformly distributed B2 CuZr micro-crystals in the glassy matrix. The latter exhibits significant plasticity and strain hardening on tension and is characterized by a fracture strength exceeding that of the parent Cu_47.5_Zr_47.5_Al_5_ glass. This study not only demonstrates superior mechanical properties of MG-matrix composites, but also offers a novel approach for their fabrication.

## Results

### Heat treatment of Cu_47.5_Zr_47.5_Al_5_ MGs

**Figure 1:**
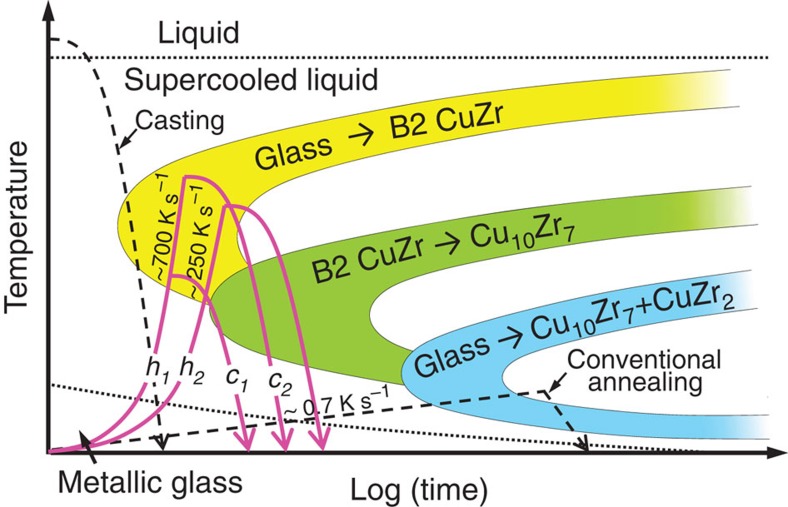
Schematic illustration of the time–temperature transformation diagram for Cu_47.5_Zr_47.5_Al_5_. Trace ‘*h*_1_' represents heating at current density *i*_1_=50±3 MA m^−2^ and trace ‘*h*_2_' represents heating at current density *i*_2_=33±3 MA m^−2^. Trace ‘*c*_1_' represents cooling starting at time *t*_1_ and trace ‘*c*_2_' represents cooling starting at time *t*_2_.

Up to date, the metallic glass-matrix composites with B2 CuZr crystals/precipitates were obtained only by rapid quenching of the melt^16^,^17^. This is schematically shown in a time–temperature transformation diagram for the Cu_47.5_Zr_47.5_Al_5_ composition in Fig. 1. Fast and adjusted cooling on solidification promotes partial crystallization of the undercooled melt into B2 CuZr and prevents its decomposition into the low-temperature Cu_10_Zr_7_ and CuZr_2_ equilibrium phases (LTEPs)^18^. So, the high-temperature metastable B2 CuZr phase, which is stable at 998–1,223 K (ref. 18), is retained at room temperature. The alternative approach to precipitate B2 CuZr can be a rapid heating of the Cu_47.5_Zr_47.5_Al_5_ (at.%) MG and then fast cooling to overcome the decomposition of B2 CuZr into LTEPs (Fig. 1).

An appropriate heating method providing a fast homogeneous and volumetric heating is Joule heating^19^,^20^. In the past, this method was shown to be effective for improvement of application-oriented physical properties of MGs^21^,^22^,^23^. Recently, Johnson *et al*.^20^ have achieved a heating rate of about 10^6^ K s^−1^ by heating a MG using a rapid capacitor discharge allowing them to ‘beat' the crystallization of the MG. However, it is difficult to control the sample temperature as well as the phase formation by capacitor-discharge heating. In the present work, we have developed and applied a method enabling *in situ* detection of crystallization, as well as control of heating rate and processing time. It is based on the measurement of the electrical resistivity of the MG, which is very sensitive to structural changes and phase formation on heating^24^. Application of this technique together with rapid Joule heating allowed to develop different composite structures by annealing Cu_47.5_Zr_47.5_Al_5_ (at.%) glassy ribbons. The obtained MG-matrix (glass+B2 CuZr) and ultrafine-structured crystal–crystal (B2 CuZr+Cu_10_Zr_7_) composites exhibit a high strength comparable to that of the parent MG together with significant tensile plastic strain.

**Figure 2:**
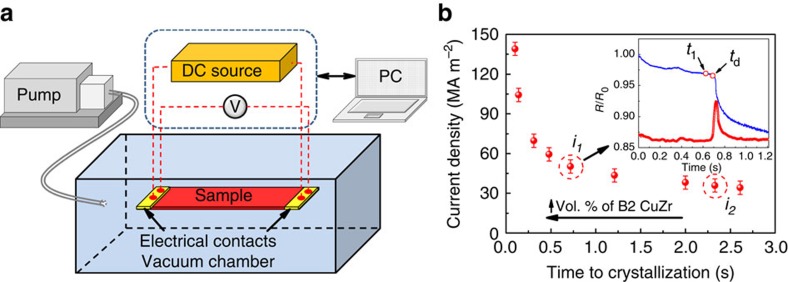
Joule heating of Cu_47.5_Zr_47.5_Al_5_ glassy ribbons. (**a**) Schematic illustration of the experimental set-up for rapid Joule heating. (**b**) Current density plotted against time until devitrification onset. The inset shows *in situ* measured normalized resistivity (lower curve) and its derivative (upper curve) corresponding to heating at current density *i*_1_=50±3 MA m^−2^. The error bars represent the s.d.

The experimental set-up for the controlled rapid heat treatment of MGs is schematically shown in Fig. 2a. A ribbon sample (25-mm long) is fixed between two electrodes placed in a vacuum chamber (∼10^−3^ mbar rest air pressure). The sample is heated by passing a high-density current through it. Simultaneously, the sample resistance is measured by the quasi four-probe method, and its time dependence (actual value and the first derivate) is *in situ* analysed by a dedicated programme. Depending on the chosen conditions (threshold value in the derivative (Fig. 2b, inset)), the current flow can be stopped and the ribbon left for cooling. A characteristic time delay between the detection of a desired threshold value and quenching is about 20 ms.

Typical ‘snapshots' of the normalized resistivity and the corresponding derivative curves recorded as a function of time at a current density *i*_1_ =50±3 MA m^−2^ are presented in the inset in Fig. 2b. The resistivity drop at *t*≈700 ms indicates the crystallization of the MG (ref. 24). It has to be noted that the resistivity drop occurs even when the current is switched off right after the specified threshold has been reached, that is, at the beginning of the resistivity drop. The reason for that is the energy generated in the ribbon during the exothermic crystallization reaction^25^, which promotes further crystallization. Since the Joule heat is proportional to the squared current density, the latter plotted against time-to-crystallization (as defined from the resistivity drop) has a non-linear behaviour (Fig. 2b). Due to a relatively short heating time, adiabatic conditions are nearly fulfilled, and it can be assumed that the current density is proportional to the heating rate. Indeed, the measurement of heating rates by a thermocouple proved that a higher current density corresponds to a higher heating rate. For instance, the heating rate corresponding to *i*_1_=50±3 MA m^−2^ and *i*_2_=33±3 MA m^−2^ is not less than 700 K s^−1^ and 250 K s^−1^, respectively.

**Figure 3:**
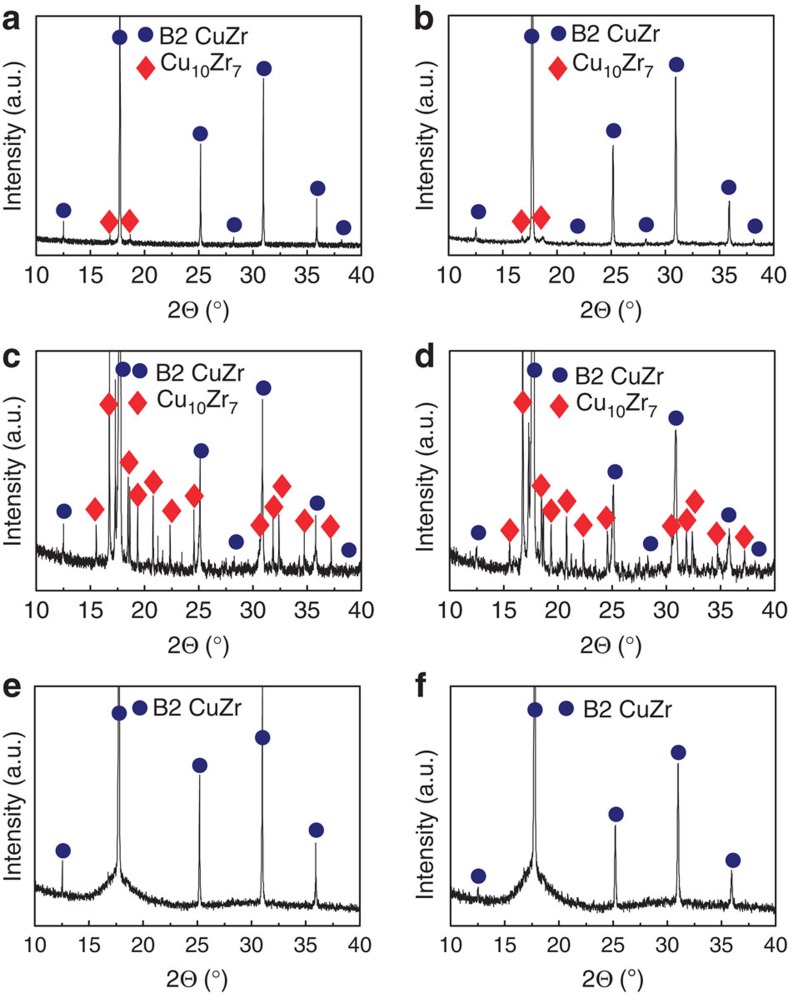
X-Ray diffraction patterns of Cu_47.5_Zr_47.5_Al_5_ ribbons after rapid Joule heating. Fully crystalline sample (FC-B2) (**a**) before and (**b**) after deformation. Ultrafine-structured composite (UFSC-B2) (**c**) before and (**d**) after deformation. Metallic glass-matrix composite (MG-B2) (**e**) before and (**f**) after deformation.

**Figure 4:**
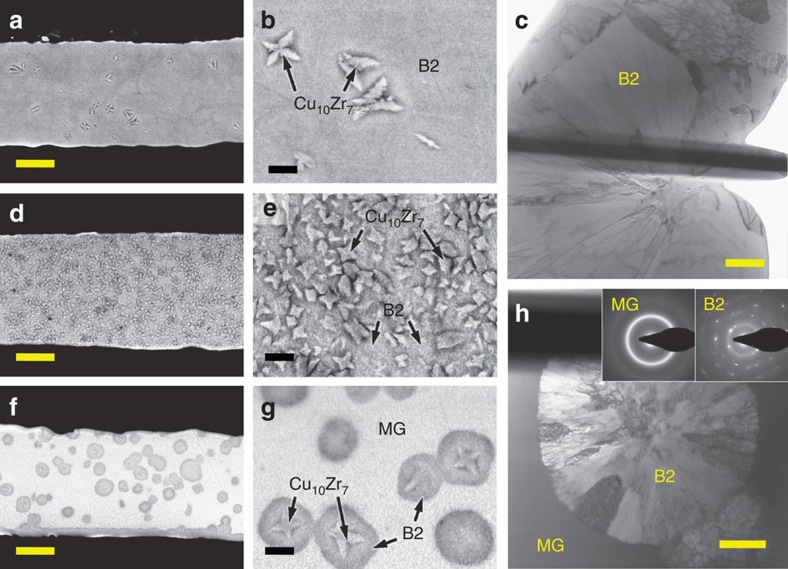
Microstructure of Cu_47.5_Zr_47.5_Al_5_ ribbons after rapid Joule heating. (**a**) SEM image of fully crystalline sample (FC-B2). Scale bar, 10 μm. (**b**) SEM image of fully crystalline sample (FC-B2). Scale bar, 2 μm. (**c**) TEM image of fully crystalline sample (FC-B2). Scale bar, 1 μm. (**d**) SEM image of ultrafine-structured composite (UFSC-B2). Scale bar, 10 μm. (**e**) SEM image of ultrafine-structured composite (UFSC-B2). Scale bar, 2 μm. (**f**) SEM image of metallic glass-matrix composite (MG-B2). Scale bar, 10 μm. (**g**) SEM image of metallic glass-matrix composite (MG-B2). Scale bar, 2 μm. (**h**) TEM image of metallic glass-matrix composite (MG-B2). Scale bar, 1 μm. Note B2, B2 CuZr phase; MG, metallic glass.

To investigate the effect of heating rate and annealing time on the microstructure, the Cu_47.5_Zr_47.5_Al_5_ glassy ribbons were heated either at different current densities (*i*_1_ and *i*_2_) until the onset of devitrification or at a constant current density but for different times (*t*_1_ and *t*_d_). X-ray diffraction (XRD) patterns as well as scanning electron microscopy (SEM) and transmission electron microscopy (TEM) images taken from representative samples are presented in Figs 3 and 4. The samples referred to as FC-B2 (fully crystalline B2 CuZr) and UFSC-B2 (ultrafine-structured composite) were obtained by heating the glassy ribbons at *i*_1_ and *i*_2_ up to the onset of devitrification (Fig. 2b). The samples denoted as MG-B2 (MG matrix with B2 CuZr phase) were obtained by heating at a constant current density *i*_1_ for the time *t*_1_=*t*_d_−50 ms (see inset in Fig. 2b).

### Microstructural and phase analysis

According to the XRD patterns, the samples annealed until the onset of crystallization are fully crystalline (Fig. 3a,c). The FC-B2 samples obtained by heat treatment at the higher current (*i*_1_) and, therefore, the higher heating rate (∼750 K s^−1^) are composed of B2 CuZr and a minor amount of Cu_10_Zr_7_ (Fig. 3a). SEM analysis revealed that the microstructure consists of the micrometre-sized Cu_10_Zr_7_ dendrites (about 4 vol.%) embedded in a B2 CuZr matrix (Fig. 4a–c). The low-temperature Cu_10_Zr_7_ phase typically forms prior to CuZr_2_^25^,^26^ and is a decomposition product of B2 CuZr as predicted by the phase diagram^18^. The average grain size of the B2 CuZr crystals, having also a fine subgrain structure, is about 9±3 μm (Fig. 4f) which is 1 order of magnitude smaller than that obtained previously for as-cast samples^16^,^17^.

The XRD pattern of the UFSC-B2 samples obtained by heat treatment at lower heating rate (∼250 K s^−1^) also shows B2 CuZr and Cu_10_Zr_7_ (Fig. 3c). However, the volume fraction of Cu_10_Zr_7_ (about 55 vol.%) is much larger than in the case of FC-B2 (Fig. 4d,e). The average size of the Cu_10_Zr_7_ dendrites (1.5±0.5 μm) embedded in the B2 CuZr matrix is about two times smaller compared with the FC-B2 samples.

The microstructural analysis of the MG-B2 samples revealed a homogeneous distribution of the B2 CuZr crystals in the glassy matrix (Fig. 4f). More detailed TEM analysis together with the selective area electron diffraction patterns also disclosed the composite microstructure of MG-B2: B2 CuZr crystals embedded in the glassy matrix (Fig. 4h). The volume fraction and the average size of B2 CuZr are about 33±3 vol.% and 5±2 μm, respectively. The B2 CuZr grains have a fine subgrain structure (Fig. 3h), and micrometre-size dendrites are found in some of them (Fig. 4g). The dendrites are assumed to be Cu_10_Zr_7_ as their morphology and composition are similar to those of the dendrites in FC-B2. To clarify the reasons of B2 CuZr formation and stabilization, we refer to Fig. 1.

**Figure 5:**
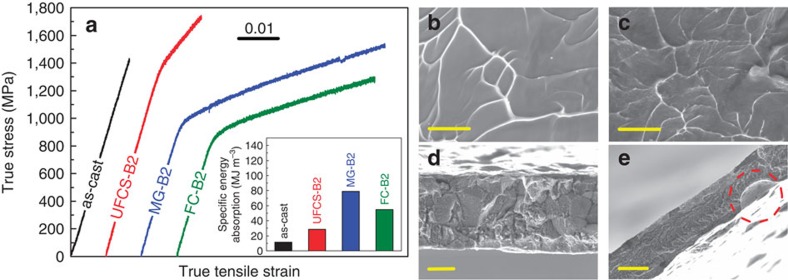
Mechanical properties at room temperature and fractography of as-cast and heat-treated Cu_47.5_Zr_47.5_Al_5_ ribbons. (**a**) Tensile properties at room temperature. The inset shows specific energy absorption values. (**b**) Fracture surface of glassy ribbons. Scale bar, 5 μm. (**c**) Fracture surface of MG-matrix composite (MG-B2). Scale bar, 5 μm. (**d**) Fracture surface of fully crystalline B2 CuZr (FC-B2). Scale bar, 10 μm. (**e**) Fracture surface of ultrafine-structured composite (UFSC-B2). Casting defect in UFSC-B2 is marked by a circle. Scale bar, 20 μm.

On conventional slow annealing at a heating rate of about 0.7 K s^−1^, Cu_47.5_Zr_47.5_Al_5_ MG decomposes into the stable Cu_10_Zr_7_ and CuZr_2_ phases^25^,^27^, as it is schematically shown in Fig. 1. In the actual rapid (actual) heat treatment, which is roughly 3 orders of magnitude faster, the polymorphous B2 CuZr phase along with the primary dendritic Cu_10_Zr_7_ phase was precipitated. It is well-known that the devitrification temperature of MGs increases with increasing heating rate^25^. According to the Al–Cu–Zr phase diagram^18^, B2 CuZr is stable at the temperature range from 998 to 1,223 K. Hence, it is likely that the rapid Joule heating of Cu_47.5_Zr_47.5_Al_5_ shifts the crystallization temperature to the temperature region, where the formation of B2 CuZr is favoured. The subsequent cooling is fast enough to prevent the full decomposition of B2 CuZr into the low-temperature equilibrium phases: only a few crystals of Cu_10_Zr_7_ were formed during the processing. Thus, one can conclude that the crystallization of the Cu_47.5_Zr_47.5_Al_5_ MG can be controlled by proper adjustment of the heat-treatment conditions. Furthermore, this enables the production of differently optimized non-equilibrium microstructures with desired, for example, mechanical, properties. This is demonstrated in Fig. 5a showing the tensile properties of as-cast and heat-treated samples.

### Mechanical properties

The heat-treated samples exhibit an improved mechanical performance, in particular, they are characterized by a remarkable plastic strain in tension (6.8±0.5%), as well as by a significant specific energy absorption (Fig. 5a, inset). The as-cast amorphous ribbons show an elastic deformation of about 1.5% but they break in a typical brittle manner without yielding on reaching a fracture stress of 1,420±50 MPa. The fracture surface exhibits characteristic vein pattern morphology (Fig. 5b). The precipitation of homogeneously distributed B2 CuZr crystals in the MG matrix has a significant impact on the tensile plastic deformation such as in MG-B2 (Fig. 5a). The high tensile plasticity of MG-B2 is due to the stabilization of shear bands by finely dispersed B2 CuZr crystals in the glassy matrix^16^. The formation and propagation of multiple shear bands on tension is evidenced by pronounced serration behaviour of the stress–strain curve for the MG-B2 sample similar to that reported for the Cu_48_Zr_48_Al_4_ MGC on compression^28^.

The yield strength of the MG-B2 composite lies between the fracture strength of the as-cast MG and the yield strength of the crystalline FC-B2 sample (Fig. 5). This is in line with the experimental and theoretical values of the yield strength for the Cu_47.5_Zr_47.5_Al_5_ composites on uniaxial compression^29^. It is worthy to note that the MG-B2 composite shows a strong strain hardening on tension and its fracture strength of 1,520±50 MPa exceeds that of the as-cast Cu_47.5_Zr_47.5_Al_5_ MG. The possible reasons for the hardening are martensitic transformation^12^, ‘blocking' effect^12^ and dislocation-mediated hardening. In contrast to previous publications^12^,^17^, XRD analysis of the MG-B2 samples before and after deformation (Fig. 3) does not show any change of their crystalline structure. However, a broadening of the X-ray diffraction peaks of deformed MG-B2 composite is indicative for a higher dislocation density. The finer vein pattern morphology of MG-B2 (Fig. 5c) in comparison with the MG samples (Fig. 5b) indicates an intensive multiplication of shear bands during deformation caused by the ‘blocking' effect. These findings suggest that the strain hardening in the MG-B2 MG-matrix composite is caused by the ‘blocking' effect and increasing dislocation density in B2 CuZr crystals.

Both fully crystalline samples (UFSC-B2 and FC-B2) exhibit high strength and pronounced tensile plasticity (Fig. 5a) comparable to the values for MG-B2. A considerable effect of the Cu_10_Zr_7_ precipitates in the B2 CuZr matrix on the mechanical properties can be revealed: the FC-B2 samples with minor volume fraction of Cu_10_Zr_7_crystals yield at 900±50 MPa and show plastic deformation of about 5.6±0.5%. This yield stress value is considerably larger than that of fully crystalline B2 CuZr (refs 16, 17). This can be ascribed to the finer grain size of the current FC-B2 samples. Unexpectedly, the plastic deformation of FC-B2 is lower compared with that of the MG-B2 composite (Fig. 5a). This is due to the weak grain boundaries in FC-B2, evidenced by the intergranular fracture of the specimen (Fig. 5d). In contrast to the fully crystalline FC-B2, there are no traces of inter phase crack propagation in the MG-B2 composite confirming the formation of a strong interface between the B2 CuZr crystals and the MG matrix (Fig. 5c).

The UFSC-B2 exhibits yielding at 1,410±50 MPa followed by significant strain hardening and plastic deformation up to a fracture stress at 1,720±50 MPa. The early failure of UFSC-B2 at 4±0.5% strain is probably due to as-cast defects (Fig. 5e). Thus, summarizing the results for both FC-B2 and UFSC-B2 fully crystalline samples, it can be concluded that the hard, but brittle Cu_10_Zr_7_ phase^30^, gives a major contribution to the strength while the tough B2 CuZr phase promotes tensile plasticity.

## Discussion

In this work, we introduced an improved Joule heating processing, which allows homogeneous, volumetric and controllable rapid heat treatment. It has been applied to glassy Cu_47.5_Zr_47.5_Al_5_ ribbons to design high-performance composite structures. The control of the heat-treatment process was carried out by *in situ* analysis of the electrical resistance of the samples. Variation of the current density (heating rate) results in formation of fully crystalline samples with different volume fraction of the B2 CuZr and Cu_10_Zr_7_ phases: the higher the applied current density the higher the volume fraction of B2 CuZr. This has been demonstrated for two selected samples, that is, the fine-grained B2 CuZr containing minor volume fraction of Cu_10_Zr_7_ (FC-B2) and ultrafine-structured B2 CuZr (UFSC-B2) containing 55 vol.% of Cu_10_Zr_7_. Optimized adjustment of the heat-treatment conditions allows obtaining uniformly distributed B2 CuZr micro-crystals in the MG matrix. For the first time a MGC with B2 CuZr dispersions was fabricated by controlled rapid heat treatment of Cu_47.5_Zr_47.5_Al_5_ MG, while before it was exclusively produced by casting. Precipitation of B2 CuZr was possible due to the shift of devitrification temperature to the high-temperature region (where formation of B2 CuZr is favoured) by rapid heating. Subsequent cooling was fast enough to prevent full decomposition of B2 CuZr into the low-temperature equilibrium phases.

The MG-matrix B2 CuZr composite exhibits high fracture strength, which is comparable with that of the parent MG. However, in contrast to the extreme brittleness of the latter, the composite shows significant tensile strain of 6.8±0.5%. The strength of a new ultrafine-structured B2 CuZr composite (UFSC-B2) even exceeds that of the parent MG. In addition, significant strain hardening supports the tensile plastic deformation of UFSC-B2, which is 4±0.5%. The designed composites exhibit several times increased specific energy absorption values compared with the parent glass. The unique mechanical performance of the new composites makes them attractive candidates for structural applications.

The presented rapid heat-treatment method can be, in general, scaled up to bulk samples as it was done in the work of Johnson *et al*.^20^, where the capacitor-discharge method was applied to bulk Vitreloy 1 glassy rods for homogeneous heating. Since the heating rate depends on current density, application of the current-controlled flash Joule annealing for bulk MGs will require an appropriate increase of the supplied power. Furthermore, to achieve a sufficient cooling of bulk samples a tool for rapid quenching, for example, similar to that reported by Johnson *et al*.^20^, has to be added. The present method can supposedly be also applied for different MGs (for example, CuZr based, Ti based) to form bulk MGCs containing the martensitic B2 phase. An optimized adjustment of the heat treatment enables tuning of the microstructure to achieve desired functional properties.

## Methods

### Sample preparation

Samples were prepared under high purity argon atmosphere in two steps. First, Cu_47.5_Zr_47.5_Al_5_ (at.%) ingots were produced from Cu (99.99%), Zr (99.98%) and Al (99.99%) by arc-melting. In the second step, glassy ribbons were prepared from ingots by melt-spinning.

### Sample characterization

The samples were characterized by XRD (STOE STADI P with Mo-K_α1_ radiation), SEM (Zeiss Leo Gemini 1530), TEM (FEI Tecnai) and ImageJ software. Phase identification was done by means of X'Pert High Score Plus software. Mechanical tests were performed with Instron 8562 machine at a strain rate of 1 × 10^−4^ s^−1^ at room temperature. The strain was measured by laser extensometer (Fiedler Optoelektronik). The gauge length of 5 mm was set in the middle region of ribbon samples.

## Additional information

**How to cite this article:** Okulov, I. V. *et al*. Flash Joule heating for ductilization of metallic glasses. *Nat. Commun.* 6:7932 doi: 10.1038/ncomms8932 (2015).
